# Dispersion of single-walled carbon nanotubes by a natural lung surfactant for pulmonary *in vitro *and *in vivo *toxicity studies

**DOI:** 10.1186/1743-8977-7-31

**Published:** 2010-10-19

**Authors:** Liying Wang, Vincent Castranova, Anurag Mishra, Bean Chen, Robert R Mercer, Diane Schwegler-Berry, Yon Rojanasakul

**Affiliations:** 1 National Institute for Occupational Safety and Health, HELD/PPRB, Morgantown, WV 26505, USA; 2 Department of Pharmaceutical Sciences, West Virginia University, Morgantown, WV 26506, USA

## Abstract

**Background:**

Accumulating evidence indicate that the degree of dispersion of nanoparticles has a strong influence on their biological activities. The aims of this study were to develop a simple and rapid method of nanoparticle dispersion using a natural lung surfactant and to evaluate the effect of dispersion status of SWCNT on cytotoxicity and fibrogenicity *in vitro *and *in vivo*.

**Results:**

The natural lung surfactant Survanta^® ^was used to disperse single-walled carbon nanotubes (SWCNT) in a biological medium. At physiologically relevant concentrations, Survanta^® ^produced well dispersed SWCNT without causing a cytotoxic or fibrogenic effect. *In vitro *studies show that Survanta^®^-dispersed SWCNT (SD-SWCNT) stimulated proliferation of lung epithelial cells at low doses (0.04-0.12 μg/ml or 0.02-0.06 μg/cm^2 ^exposed surface area) but had a suppressive effect at high doses. Non-dispersed SWCNT (ND-SWCNT) did not exhibit these effects, suggesting the importance of dispersion status of SWCNT on bioactivities. Studies using cultured human lung fibroblasts show that SD-SWCNT stimulated collagen production of the cells. This result is supported by a similar observation using Acetone/sonication dispersed SWCNT (AD-SWCNT), suggesting that Survanta^® ^did not mask the bioactivity of SWCNT. Likewise, *in vivo *studies show that both SD-SWCNT and AD-SWCNT induced lung fibrosis in mice, whereas the dispersing agent Survanta^® ^alone or Survanta^®^-dispersed control ultrafine carbon black had no effect.

**Conclusions:**

The results indicate that Survanta^® ^was effective in dispersing SWCNT in biological media without causing cytotoxic effects at the test concentrations used in this study. SD-SWCNT stimulated collagen production of lung fibroblasts *in vitro *and induced lung fibrosis *in vivo*. Similar results were observed with AD-SWCNT, supporting the conclusion that Survanta^® ^did not mask the bioactivities of SWCNT and thus can be used as an effective dispersing agent. Since excessive collagen production is a hallmark of lung fibrosis, the results of this study suggest that the *in vitro *model using lung fibroblasts may be an effective and rapid screening tool for prediction of the fibrogenic potential of SWCNT *in vivo*.

## Background

Advances in nanotechnology have made possible the fabrication of materials at the nanoscale level. Carbon nanotubes (CNT) are a major class of nanomaterials possessing unique mechanical, electrical, and thermal properties. As the use of CNT has become more widespread, there has been a great concern about their potential adverse effects on human health and the environment. Nanoparticles can come in contact with the human body through inhalation as well as ingestion and dermal deposition. Pulmonary exposure could occur due to aerosolization of nanomaterials including agglomerates of different size and shape. Individual CNT have a very high aspect ratio and can agglomerate into structures which are micrometers in diameter in the dry state or upon suspension in polar and non-polar solvents [1-4]. Animal exposure studies have shown that a major pathologic effect of CNT exposure is pulmonary fibrosis, and that this effect is dependent on the physiochemical properties of CNT [5-7]. The dispersion status of single-walled carbon nanotubes (SWCNT) has been shown to influence deposition pattern as well as biological effect [5,6]. Large agglomerates deposit in the proximal alveoli and induce granulomas. In contrast, more dispersed structures can deposit in the distal alveoli, rapidly migrate into the alveolar walls, and induce interstitial fibrosis.

To aid the investigations of pulmonary responses to nanoparticle exposure, several *in vitro *and *in vivo *models have been developed. These studies often rely on the use of nanoparticle preparations suspended in physiological solutions. Since nanoparticles in solution tend to form coarse agglomerates in physiological media, development of methods to disperse nanoparticles is important in assessing their biological activities. Over the years, a variety of methods have been described to disperse nanoparticles, including the use of cell culture reagents [8,9], dimethyl sulfoxide [10], acetone [5], pluronic surfactant [11], Tween 80 [12], and pulmonary lavage fluids [13]. Some of these methods are laborious, time consuming, potentially toxic, and may not mimic physiological condition. Therefore, the aim of this study was to develop a simple, rapid, and safe method of nanoparticle dispersion using the natural lung surfactant Survanta^® ^for *in vitro *and *in vivo *studies. Survanta^® ^is a surfactant replacement used by health care professionals for prevention and treatment of respiratory distress syndrome in premature infants. It is a sterile product consisting of phospholipids and surfactant-associated proteins SP-B and SP-C. Its commercial availability, biocompatibility and safety make this preparation an attractive dispersing agent for biological studies of nanoparticles. In the present study, we evaluated the ability of Survanta^® ^to disperse SWCNT and investigated the bioactivity of dispersed SWCNT *in vitro *and *in vivo*. We also compared the effect of Survanta^®^-dispersed SWCNT to SWCNT dispersions prepared by previously published acetone/sonication and aerosolization methods [5,7,14].

## Results

### Nanoparticle dispersion by Survanta^®^

**Figure 1:**
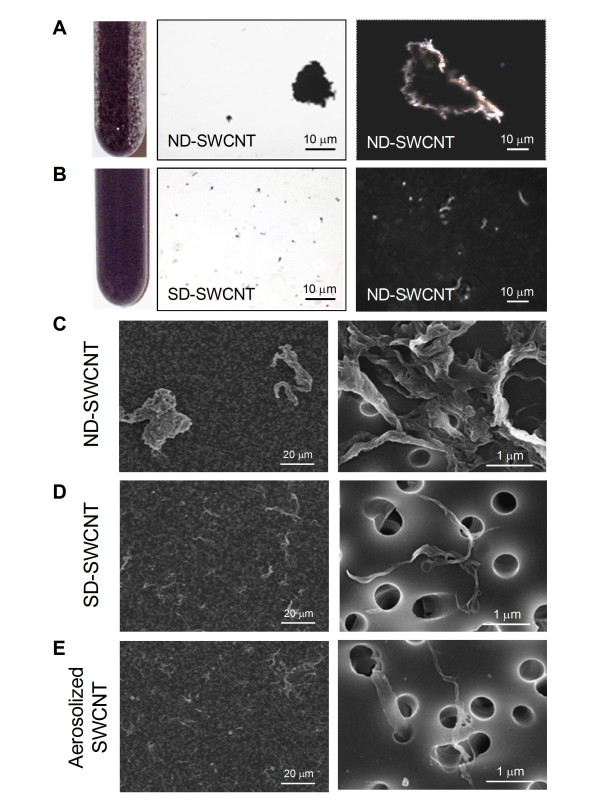
**Comparison of Survanta**^**^®^**^**-dispersed and non-dispersed SWCNT**. (A) The non-dispersed SWCNT suspension (0.1 mg/ml in PBS) shows visible clumping of the SWCNT (left panel) with corresponding light microscopy (middle panel, 100x) and hyperspectral imaging of an individual clump (right panel, 400x). (B) The Survanta^®^-dispersed SWCNT suspension at the same concentration shows much improved dispersion with no visible large clumps (left panel). Corresponding light microscopy (middle panel) and hyperspectral imaging (right panel) show a uniform dispersion of the particles. (C-E) Field emission scanning electron microscopy of non-dispersed, Survanta^®^-dispersed, and aerosolized SWCNT at low magnification (400x, left panel) and high magnification (30,000x, right panel). Aerosolization of SWCNT was performed according to the method previously described by our group [14].

**Figure 2:**
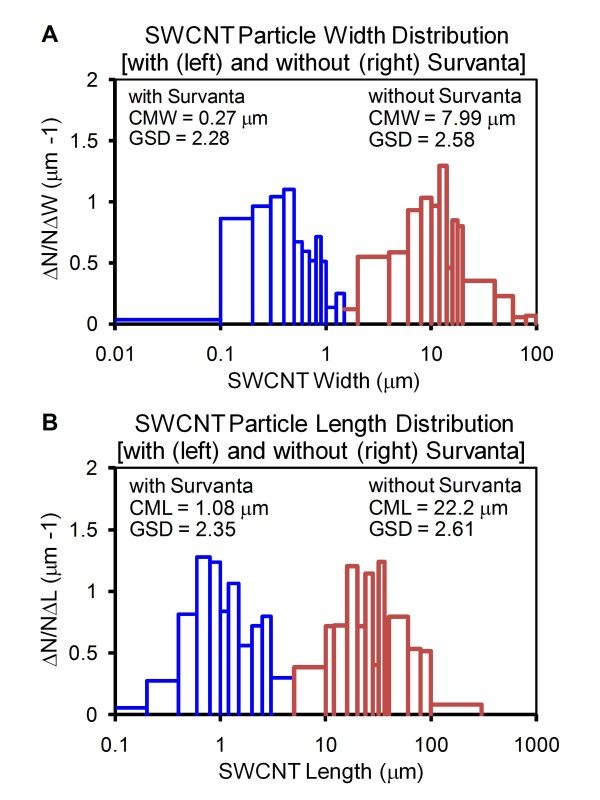
**Effect of Survanta**^**^® ^**^**on SWCNT particle size and distribution**. SWCNT were dispersed in PBS in the presence or absence of Survanta^® ^as described in Figure 1. (A) Width distribution of non-dispersed and Survanta^®^-dispersed SWCNT. (B) Length distribution of non-dispersed and Survanta^®^-dispersed SWCNT. CMW = count median width; CML = count median length; GSD = geometric standard deviation. The size and distribution values were determined from triplicate experiments with each experiment measuring a minimum of 300 particles.

Visual inspection and corresponding micrographs of the non-dispersed SWCNT (ND-SWCNT) and Survanta^®^-dispersed SWCNT (SD-SWCNT) are shown in Figure 1. The concentration of 150 μg/ml of Survanta^® ^used in this preparation is based on the content of lung surfactants found in rodent lung lavage fluids [13]. Micrometer SWCNT agglomerates were noted in ND-SWCNT suspension (Figure 1A). In contrast, Survanta^® ^dispersed SWCNT into smaller size structures as shown by visual inspection (Figure 1B, left panel) or by regular light and hyperspectral microscopy (Figure 1B, middle and right panels). Field emission scanning electron micrographs and count-mode particle size analysis of ND-SWCNT and SD-SWCNT suspended structures are shown in Figure 1 C and 1D comparing to aerosolized SWCNT (Figure 1E) and Table 1, respectively. SD-SWCNT showed suspended structures of a smaller size, exhibiting count median width (CMW) and count median length (CML) of 0.3 μm × 1 μm, compared to ND-SWCNT with 8 μm (CMW) × 22 μm (CML), respectively (Figure 2). These results indicate that Survanta^® ^substantially improved the dispersion of SWCNT to such a degree that is comparable with the structure sizes reported for aerosolization of dry SWCNT [14].

**Table 1:** Size of non-dispersed and Survanta^®^-dispersed particles

	Width* (μm)	Length* (μm)
SD-SWCNT	0.38 ± 0.02	1.42 ± 0.08

ND-SWCNT	12.35 ± 0.76	27.72 ± 1.86

SD-UFCB	0.70 ± 0.04	0.93 ± 0.05

ND-UFCB	5.07 ± 0.18	6.54 ± 0.2

* mean ± SD, n = 300

Table 1 gives the average diameter and length of SD-SWCNT *vs*. ND-SWCNT. The mean width of SD-SWCNT was 380 nm. About two-thirds of the SD-SWCNT were dispersed into structures with diameters less than the average diameter of 380 nm, and more than 8% of the dispersed particles were less than 100 nm in diameter (Table 2). In contrast, no particles with less than 380 nm in diameter were observed in ND-SWCNT preparations (Table 2). These results indicate that Survanta^® ^substantially improves the dispersion of SWCNT at the concentration used.

**Table 2:** Percentage of well dispersed particles in suspension

	% with width of <0.38 μm*	% with width of <0.1 μm
SD-SWCNT	65.6%	8.6%

ND-SWCNT	0%	0%

	% with width of <0.70 μm^#^	% with width of <0.1 μm

SD-UFCB	73.0%	1.1%

ND-UFCB	0%	0%

* average width of SD-SWCNT; # average width of SD-UFCB

### Effect of Survanta^® ^on cytotoxicity

**Figure 3:**
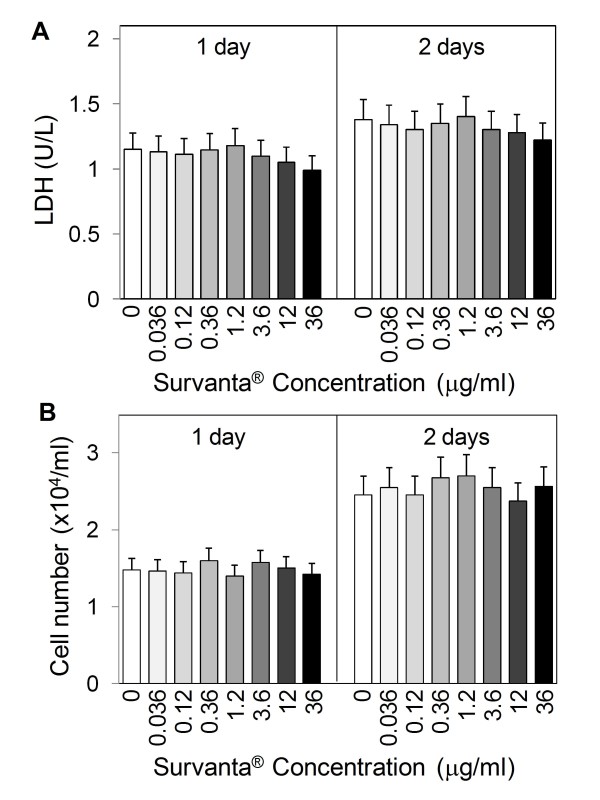
**Effect of Survanta**^**^® ^**^**on cell toxicity and cell number**. Subconfluent (80%) cultures of human lung epithelial BEAS-2B cells were exposed to various concentrations of Survanta^® ^and, at one and two days after the treatment, cells were analyzed for cytotoxicity and cell number by LDH assay (A) and cell counting (B). Plots are mean ± S.D. (n = 4). No significant changes over non-treated controls were observed in all measurements at *p *< 0.05.

To be useful as a dispersion agent for nanoparticles, Survanta^® ^should exhibit no cytotoxic effect and should not mask the bioactivity of nanoparticles. We first tested the cytotoxic effect of Survanta^® ^alone in the absence of SWCNT on lung epithelial cells by lactate dehydrogenase (LDH) assay and by direct cell count. The results show that at the concentrations tested (0.036-36 μg/ml), Survanta^® ^had no significant effects on the LDH release and cell number as compared to non-treated control (Figure 3 A and 3B), suggesting its biocompatibility at the test concentrations.

### Effects of Survanta^®^-dispersed SWCNT on cell growth and toxicity

**Figure 4:**
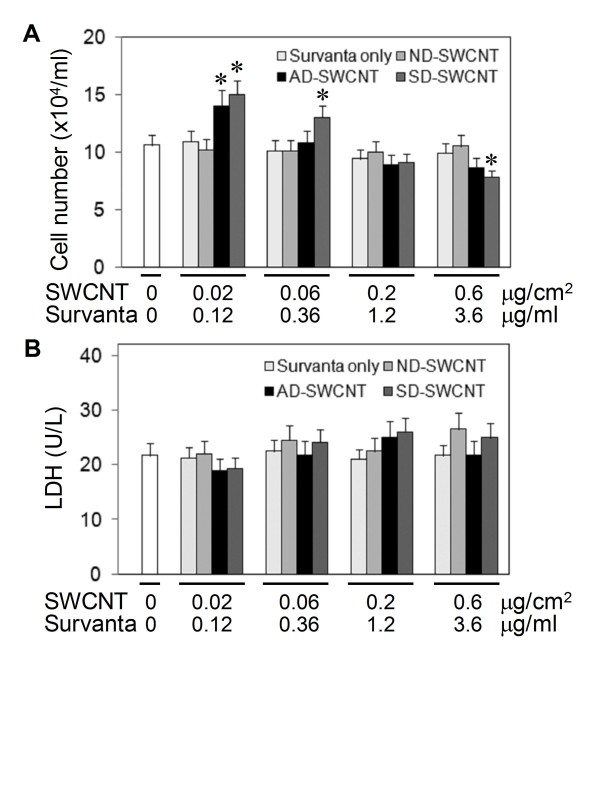
**Effect of SWCNT on cell proliferation and cell viability**. Subconfluent (80%) cultures of lung epithelial BEAS-2B cells were either left untreated or treated with the indicated concentrations of non-dispersed (ND-), Survanta^®^-dispersed (SD-), or acetone/sonication-dispersed (AD-) SWCNT, or Survanta^® ^alone, or no treatment (No Tx) for 24 h. (A) Cell proliferation was determined by hemocytometry. (B) Cell toxicity was determined by LDH assay. Plots are mean ± S.D. (n = 4). *p < 0.05 versus non-treated control.

We next evaluated the effects of SWCNT, dispersed or non-dispersed by Survanta^®^, on cell toxicity and proliferation. Figure 4A shows that Survanta^®^-dispersed SWCNT exhibited a growth stimulating effect at low doses (0.02 and 0.06 μg/cm^2 ^or 0.04 and 0.12 μg/ml) and suppressing effect at the highest dose tested (0.6 μg/cm^2 ^or 1.2 μg/ml), whereas non-dispersed SWCNT had no effect on cell proliferation as compared to non-treated or Survanta^® ^only treated control. These results suggest the importance of dispersion status of SWCNT on their bioactivity, which is supported by the observation that SWCNT dispersed by acetone/sonication (AD-SWCNT) also induced cell proliferation at the lowest dose tested (Figure 4A). These data suggest that Survanta^® ^did not mask the bioproliferative activity of SWCNT. LDH studies show that SD-SWCNT and other test agents were non-toxic at all concentrations tested (Figure 4B). These results indicate that non-cytotoxic doses of dispersed SWCNT can alter the growth pattern of human lung epithelial cells. To our knowledge, this is the first demonstration of the proliferative effect of low-dose SWCNT on lung epithelial cells.

### Effect of dispersed SWCNT on collagen production by lung fibroblasts

**Figure 5:**
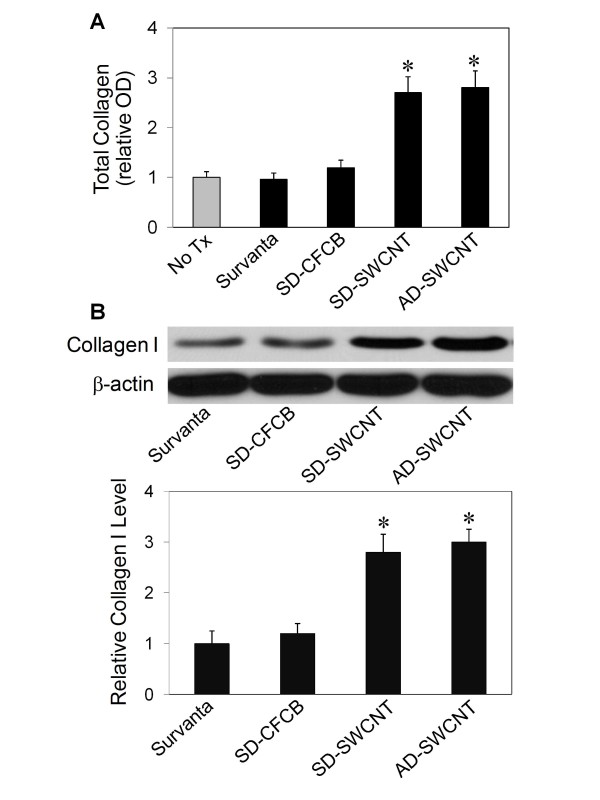
**Effect of SWCNT on fibroblast collagen production**. Subconfluent cultures of human lung fibroblast CRL-1490 cells were either left untreated (No Tx) or treated with Survanta^® ^alone, Survanta^®^-dispersed SWCNT (SD-SWCNT), Survanta^®^-dispersed ultrafine carbon black (SD-UFCB), or acetone/sonication-dispersed SWCNT (AD-SWCNT) at a particle concentration of 0.02 μg/cm^2 ^or 0.04 μg/ml. At 2 days after the treatment, cells were washed, lysed, and analyzed for total collagen content by the Sircol^® ^assay (A) or collagen I expression by Western blotting (B), as described in the *Methods *section. Equal amounts of total protein per sample were used in all measurements. β-actin was used as a loading control for Western blotting. The blot signals were quantified by densitometry and mean data from independent experiments (one of which is shown here) were normalized to the result obtained in cells without treatment. Plots are mean ± S.D. (n = 4). *p < 0.05 versus non-treated control.

Fibrosis is a fibroproliferative disorder characterized by overproduction and accumulation of extracellular matrix, notably collagens [5,14]. We tested whether SD-SWCNT can stimulate collagen production by lung fibroblasts in culture. AD-SWCNT which have been shown to induce interstitial lung fibrosis [5] were used as a positive control. Ultrafine carbon black (UFCB) dispersed in Survanta^® ^was used as a negative particle control. The results show that as compared to non-treated control, Survanta^® ^alone or SD-UFCB had no significant effect on cellular collagen content, as determined by Sircol^® ^assay which detects total collagen content (Figure 5A). In contrast, SD-SWCNT and AD-SWCNT caused a substantial increase in cellular collagen content at 0.02 μg/cm^2 ^(0.04 μg/ml). Western blot analysis of collagen I, which is the most abundant collagen in the lung [15], shows a similar collagen-inducing effect of SD-SWCNT and AD-SWCNT, whereas Survanta^® ^alone and SD-UFCB had a minimal effect (Figure 5B). These results indicate that SD-SWCNT was able to stimulate collagen production in lung fibroblasts and that Survanta^® ^used for the SWCNT dispersion had no interfering or masking effect on the collagen-inducing activity of SWCNT, i.e., as compared to AD-SWCNT.

### Effect of dispersed SWCNT on lung fibrosis in mice

**Figure 6:**
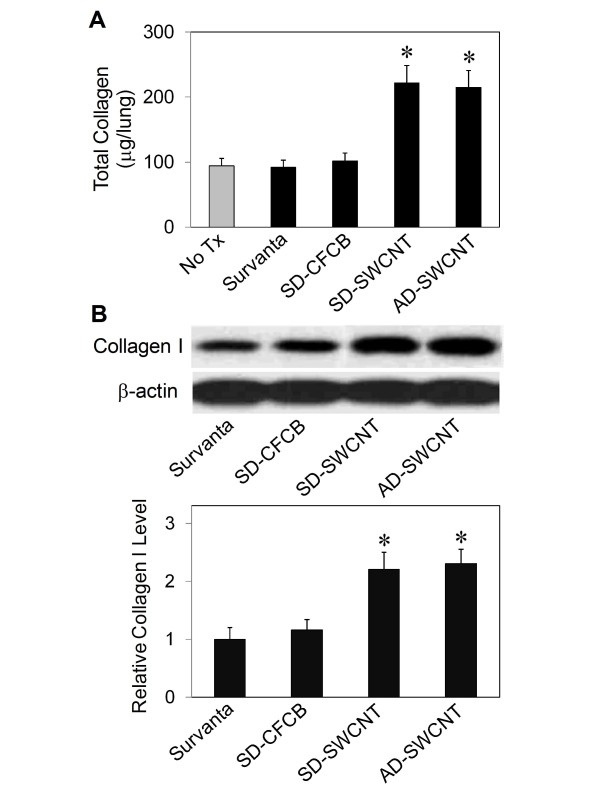
**Effect of SWCNT on lung fibrosis in mice**. Mice were pharyngeal aspirated with Survanta^® ^alone, Survanta^®^-dispersed SWCNT (SD-SWCNT) or acetone/sonication-dispersed SWCNT (AD-SWCNT), or Survanta^®^-dispersed ultrafine carbon black SD-UFCB) at the particle concentration of 10 μg/mouse. At 2 weeks after the treatment, mice were sacrificed and the lungs were isolated, lysed, and analyzed for collagen content by the Sircol^® ^assay (A) and Western blotting (B). Equal amounts of total protein per sample were used. β-actin was used as a loading control for Western blotting. The blot signals were quantified by densitometry and mean data from three independent experiments were normalized to the result obtained in cells without treatment. Plots are mean ± S.D. (n = 3). *p < 0.05 versus non-treated control (No Tx).

To evaluate the *in vivo *fibrogenic effect of SWCNT, mice were treated with 10 μg/mouse, (~0.02 μg/cm^2 ^of alveolar epithelial surface area in the mouse lung) SD-SWCNT or control treatments, and analyzed for lung collagen content at two weeks post-treatment. Figure 6A shows that both SD-SWCNT and AD-SWCNT were able to increase lung collagen content as determined by Sircol^® ^assay, whereas SD-UFCB or Survanta^® ^only treatment had no significant effect as compared to non-treated control (No Tx). Similar results were observed with collagen I content as determined by Western blot assay (Figure 6B), supporting the fibrogenic effect of SWCNT. These results are consistent with the *in vitro *data and indicate the fibrogenic effect of SWCNT and lack of this effect by Survanta^® ^alone. The observed similarity of the fibrogenic effect of SD-SWCNT and AD-SWCNT indicates that Survanta^® ^did not mask the bioactivity of SWCNT.

## Discussion

The major goal of this study was to evaluate the suitability of utilizing Survanta^® ^as a dispersing agent for SWCNT to aid study of their bioactivity. The rationale behind its use include its simplicity and rapidity of nanoparticle dispersion (i.e., it is a single step process), biocompatibility (i.e., it has been approved for human clinical use), and commercial availability particularly as a sterile preparation which greatly facilitates *in vivo *and *in vitro *studies that require sterile conditions. In addition, one would argue that inhaled SWCNT would initially interact with alveolar lining fluid, which is modeled by Survanta^® ^suspension. However, the effectiveness of this preparation in dispersing nanoparticles and its possible interfering effect on the bioactivities of SWCNT are not known, and, therefore, are investigated in the present study. The data presented demonstrate that Survanta^® ^when used at the indicated concentrations is effective in dispersing SWCNT, yielding nanoparticles with dimensions similar to those observed after aerosolization of dry SWCNT or acetone/sonication dispersion of SWCNT [5,14]. Non-dispersed SWCNT form large agglomerates in phosphate-buffered saline (PBS) with an average width of 12.35 μm and an average length of 27.72 μm. In contrast, Survanta^®^-dispersed SWCNT form much smaller structures with an average width of 0.38 μm and an average length of 1.42 μm. The majority of the dispersed SWCNT is in the form of small bundles with no or minimum detectable individual nanotubes. The reported average diameters of aerosolized dry SWCNT and acetone/sonication-dispersed SWCNT are 0.24 μm and 0.6 μm, respectively [5,14]. These results are in good agreement with previous reports showing count median aerodynamic diameter of dry CNT generated aerosols for inhalation studies [14,16], structure size distribution found in the workplace [17], and CNT structures produced by surfactant dispersion [18]. The data indicate that aerosolized dry SWCNT and surfactant-dispersed SWCNT form much smaller aggregates than SWCNT suspended in PBS. Previous animal studies also showed that well-dispersed SWCNT after pulmonary administration exhibited similar deposited structures and dimensions (e.g., submicron-sized aggregates) as those observed in this study [5].

Ideally, *in vivo *pulmonary exposure studies should be performed using an inhalation method, since it best mimics the human exposure condition. However, the need for specialized facilities and equipment, trained personnel, and large quantities of nanoparticles has limited the use of this technology. Pulmonary aspiration represents an alternative method that has proven useful in many pulmonary toxicity studies. This method is simple, economical, uses small amounts of nanoparticles, and provides deep lung deposition as well as high correlation to the administered dose [19]. Recent studies by our group have shown that CNT administered by this method produced pulmonary fibrosis in lab animals similar to that observed after inhalation of CNT [5,6,14]. These studies suggest that the aspiration method is a reasonable alternative method to inhalation for the study of pulmonary fibrosis induced by nanoparticles. Present SWCNT-size analysis further supports the aspiration method using SD-SWCNT in which the average diameter is comparable with aerosolized dry SWCNT using for inhalation study.

The use of Survanta^® ^for nanoparticle dispersion provides an additional advantage over other methods of dispersion for pulmonary studies as it better mimics the natural lung condition. This is particularly important for *in vitro *studies which normally lack lung surfactants that could have an effect on cell interaction and bioactivity of nanoparticles. Previous studies have shown that lung surfactants aid in the displacement of particles from air to the aqueous phase and towards the lung epithelium [19]. In addition, when particles are present in peripheral airways and alveoli they exist in a completely immersed, wetted state below the surfactant film [20]. These studies suggest that experiments using lung surfactants may be more physiologically relevant than non-surfactant systems.

A key concern about the use of Survanta^® ^is its possible adverse effects on cells and tissues or masking effect on the exposed particles [21,22]. Our results show that Survanta^®^, when used at the indicated concentrations, had no significant cytotoxic effect on lung cells *in vitro *and did not induce collagen production or mask the fibrogenic effect of SWCNT either *in vitro *or *in vivo*. The results of this study also indicate that dispersion status of SWCNT is a key determinant of its biological activities to induce cell proliferation and enhance collagen production.

Another key finding of this study is the correlation between *in vitro *and *in vivo *fibrogenic responses to SWCNT and control particles under different dispersion conditions. This finding suggests the potential utility of *in vitro *lung fibroblasts as a predictive model for *in vivo *fibrogenicity testing of CNT and other nanomaterials. Fibrogenicity testing of nanomaterials is usually performed using animals. However, this method of testing is time-consuming, laborious, and costly. This combined with the rapid growth in nanotechnology, which produces an uncountable number and variety of nanomaterials, makes it impractical to test all of these materials using animals. The *in vitro *model described here represents an alternative method that could serve as a rapid screening tool for fibrogenicity testing of a large number of nanomaterials. This model can also be used to conduct detailed mechanistic studies of the fibrogenic effect of nanoparticles, which may not be achievable *in vivo*.

## Conclusions

The present study describes a novel method of CNT dispersion using the natural lung surfactant Survanta^®^. Survanta^® ^was shown to be effective in dispersing SWCNT and caused no cytotoxic or fibrogenic effect to the test lung cells under the experimental conditions. SD-SWCNT and AD-SWCNT similarly stimulated collagen production of lung fibroblasts *in vitro *and both induced lung fibrosis *in vivo*, indicating the fibrogenicity of SWCNT and non-masking effect of Survanta^®^. The reported *in vitro *cell model system could potentially be used to aid the fibrogenicity testing of CNT of various size and functionalization as well as mechanistic studies of other nanoparticles.

## Methods

### Particles

SWCNT (CNI, Houston, TX) were produced by the high pressure CO disproportionation (HiPco) technique, employing CO in a continuous-flow gas phase as the carbon feedstock and Fe(CO)_5 _as the iron-containing catalyst precursor. These SWCNT were then purified by acid treatment to remove metal contaminates for use in this study. Elemental analysis of the supplied SWCNT by nitric acid dissolution and inductively coupled plasma-atomic emission spectrometry (ICP-AES, NMAM #7300) showed that the SWCNT were 99% elemental carbon and 0.23% iron. The specific surface area was measured at -196°C by the nitrogen absorption-desorption technique (Brunauer Emmet Teller method, BET) using a SA3100 Surface Area and Pore Size Analyzer (Beckman Coulter, Fullerton, CA). The diameter and length distribution of poorly and well-dispersed preparations of SWCNT (without or with Survanta^®^) were measured by field emission scanning electron microscopy. The surface area of dry SWCNT was 400-1,000 m^2^/g, and the length and width of individual (dry) SWCNT were 0.1-1 μm and 0.8-1.2 nm, respectively. Characterization studies were performed at NIOSH research facilities as previously described [23]. The same lot of SWCNT was used for all experiments.

### Particle dispersion

SWCNT were dispersed by using Survanta^® ^(Abbott Laboratories, Columbus, OH) or by the acetone-sonication method as described previously [5]). Survanta^®^-dispersed SWCNT (SD-SWCNT) were prepared by dispersing SWCNT (0.1 mg/ml) in PBS containing Survanta^® ^(150 μg/ml) with light sonication (Sonic Vibra Cell Sonicator, Sonic & Material Inc, Newtown, CT, USA) at a power of 130 W, frequency of 20 kHz, and amplitude settings of 60% for 5-10 seconds. Non-dispersed SWCNT (ND-SWCNT) were prepared similarly but in the absence of Survanta^®^. Acetone/sonication dispersed SWCNT (AD-SWCNT) were prepared according to the method previously described [5]. Briefly, SWCNT were treated with acetone and placed in an ultrasonic bath for 24 h. The dispersed CNT were then filtered from the solution using a 20-μm nylon mesh screen followed by a 0.2-μm polytetrafluoroethylene filter. After filter collection, the dispersed CNT were washed thoroughly with distilled water and suspended in PBS with 2-3 minute sonication (Sonic Vibra Cell Sonicator, Sonic & Material Inc, Newtown, CT, USA).

### Particle imaging and size measurements

Images of SWCNT suspensions were obtained by field emission scanning electron microscopy and nanoscale hyperspectral microscopy. To assess the size distribution of SWCNT samples, a sample of each was taken and filtered through a polycarbonate filter (VCTP02500 isopore membrane; Millipore, Billerica, MA) to collect the particles. After washing with water and drying, the filter was cut into equal sections, mounted onto aluminum stubs with double-stick carbon tape, and sputter coated with gold/palladium. The deposited particles were viewed under a field emission scanning electron microscope (model S-4800; Hitachi, Tokyo, Japan) at 400 and 30,000 magnifications. The average length and width of the particles in each sample were determined by analysis of a minimum of 300 particles. The size and distribution values were determined from triplicate experiments. Representative micrographs of the SWCNT samples were taken using conventional and hyperspectral microscopy. The latter system (CytoViva, Auburn, AL) is capable of identifying specific material at a sub 100-nanometer resolution based on the material's unique spectral signature. Hyperspectral images of SWCNT were captured with the CytoViva spectrophotometer and an integrated CCD camera mounted on an Olympus BX-51 microscope at 400x.

### Cell growth and cytotoxicity

Human lung epithelial BEAS-2B cells (American Type Culture Collection, Manassas, VA) were incubated in a 24-well plate at the density of 2 × 10^4 ^cells/well in Dulbecco's modified eagle medium containing 5% fetal bovine serum. The cells were treated with various concentrations of Survanta^®^, ND-SWCNT, SD-SWCNT, or AD-SWCNT at 37°C. At the indicated times after the treatment, cell supernatants were collected and analyzed for lactate dehydrogenase (LDH) as an indicator of cell toxicity. LDH activity was determined by LDH-catalyzed oxidation of pyruvate coupled with the reduction of NAD at 340 nm using a commercial assay kit and a Cobas Mira Plus transfer analyzer (Roche Diagnostics System, Montclair, NJ). Cell growth was determined by direct cell counting of the control and treated cells. The cells were trypsinized, suspended in 100 μl culture medium, and 10 μl samples of the suspension were mixed with trypan blue for cell number counting and determination of cell viability using a hemocytometer.

### Collagen assays

Collagen content was determined by Western blotting and the Sircol^® ^assay (Biocolor Ltd., Belfast, UK). Human lung fibroblast CRL-1490 cells (ATCC, Manassas, VA) or mice were treated with Survanta^®^, SWCNT, or control particles as described below. Treated cells or mouse lung tissues were lysed and cell/tissue lysates were analyzed for protein content using a bicinchoninic acid protein assay kit (Pierce Biotechnology, Rockford, IL). For Western blot analysis, equal amounts of protein per sample (25 μg) were resolved by 10% sodium dodecyl sulfate-polyacrylamide gel electrophoresis (SDS-PAGE) and transferred onto a nitrocellulose membrane. The membrane was blocked with T-PBS (0.3% Tween-20 in PBS) containing 3% dry milk and incubated with primary antibodies specific for collagen type I and β-actin (Fitzgerald, Concord, MA) at 4°C overnight. After three washes with T-PBS, the membrane was incubated with peroxidase-conjugated secondary antibody for 1 h and then washed with 0.05% Tween-20 in PBS. The immune complexes were detected by chemiluminescence (Supersignal^® ^West Pico, Pierce, Rockford, IL) and quantified using analyst/PC densitometry software (Bio-Rad Laboratories, Hercules, CA).

For analysis of collagen content by the Sircol^® ^assay, cell/tissue lysates (50 μl) were incubated with Sirius red reagent (50 μl) for 30 min, after which the collagen-dye complex was precipitated by centrifugation at 16,000 g for 5 min. The precipitates were washed with ethanol and dissolved in 0.5 M NaOH. The samples were then introduced into a microplate reader and absorbance determined at 540 nm.

### Animal exposure

Pathogen-free male C57BL/6J mice (Jackson Laboratories, Bar Harbor, ME) weighing 25-30 grams were used. The animals were individually housed in an Association for Assessment and Accreditation of Laboratory Animal Care-accredited facility and allowed to acclimate at least 1 week prior to use. All experimental procedures were conducted in accordance with a protocol approved by the NIOSH Institutional Animal Care and Use Committee. The animals were treated with the test materials by pharyngeal aspiration as described previously [24]. Briefly, animals were anesthetized by an intraperitoneal injection of ketamine and xylazine (45 and 8 mg/kg) and placed on a board in the supine position. The animal's tongue was extended with padded forceps. A suspension of the test material (10 μg/100 ml per mouse) was placed on the back of the tongue. A slight pull of the tongue results in a reflex gasp and aspiration of the droplet. The tongue was held, and the animal was monitored for a few breaths after aspiration. All mice survived the pharyngeal aspiration procedure. At given post-exposure times, mice were sacrificed and lung tissues were isolated, homogenized, lysed and analyzed for collagen content by Western blot and Sircol^® ^assays.

### Statistics

Data were analyzed by ANOVA (STATGRAF). Bartlett's test was used to test for homogeneity of variances between groups. Statistical differences were determined by one-way ANOVA, with significance set at *P *< 0.05. When significant F-values were obtained, individual means were compared with control using a two-sided Dunnett's test. *P *< 0.05 was considered to be significant. Data are given as means ± SD. The size distributions of SWCNT particles in different media were determined using the procedures described in Hinds [25].

## Competing interests

The authors declare that they have no competing interests.

## Authors' contributions

LW conceived the study, carried out *in vitro *and *in vivo *experiments, and drafted the manuscript. VC participated in the design of the study, evaluation of results, and helped to draft the manuscript. AM participated in *in vitro *studies and Western blot analysis. BC participated in particle measurements and data analysis. RM participated in animal exposure studies. DS-B carried out the electron microscopy. YR participated in study design, particle dispersion, collagen assays, and manuscript preparation. All authors read and approved the final manuscript.
